# Resveratrol protects male *Drosophila melanogaster* from heat stress-induced paralysis through a mechanism independent of heat shock response activation

**DOI:** 10.17912/micropub.biology.001793

**Published:** 2025-10-10

**Authors:** Nichole Webb, Riley Bricker, Tyra Skalos, Annette Choi, Austin Shirk, Stacy L. Hrizo, Martin S. Buckley

**Affiliations:** 1 Biology, Slippery Rock University

## Abstract

There is great interest in understanding the mechanism of action of antioxidant treatments and their cellular effects. Heat shock causes proteins to denature and lose their function. To mitigate this, cells utilize the heat shock response (HSR) pathway, which produces Hsp70 chaperone protein to refold misfolded proteins. Heat shock also generates reactive oxygen species (ROS) that can damage cellular proteins. Here, we show that the dietary antioxidant resveratrol slows heat shock-induced paralysis in male *Drosophila melanogaster* . Live-cell imaging and molecular assays reveal this effect is not due to resveratrol activating the HSR prior to stress, suggesting involvement of ROS reduction or alternative stress pathways.

**Figure 1. Resveratrol delays heat stress-induced paralysis without activating the heat shock response:**
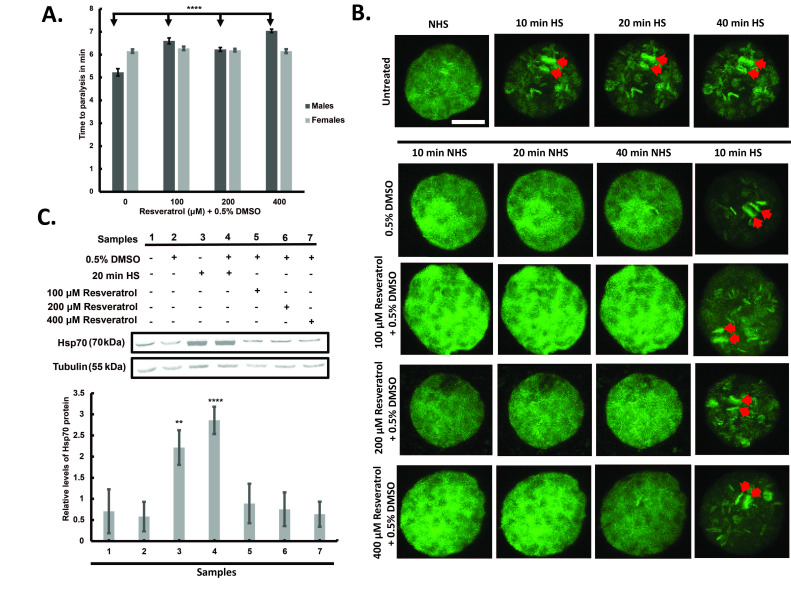
A.) Wild-type Canton-S *Drosophila melanogaster* males and females were fed 0.5% DMSO (vehicle control) or resveratrol (100 µM, 200 µM, or 400 µM in 0.5% DMSO) for five days prior to heat stress at 39°C **.** The average time to paralysis is shown for each condition. Each treatment group consisted of 10 flies per replicate, with three independent replicates (n = 30 per condition). Error bars represent 95% confidence intervals. One-way ANOVA revealed a significant treatment effect in males, with resveratrol significantly delaying paralysis at all concentrations compared to untreated controls (****p < 0.0001, Tukey’s post hoc test). No significant differences were observed in females. B.) Maximum intensity confocal microscopy images are shown for representative salivary gland nuclei from male transgenic GFP-HSF *Drosophila melanogaster* under different treatment conditions. From top to bottom, the panel shows the untreated positive control group (no DMSO, no resveratrol), the vehicle control (0.5% DMSO), and the resveratrol-treated groups (100 µM, 200 µM, and 400 µM resveratrol in 0.5% DMSO). For the positive control, nuclei were imaged under non-heat shock (NHS) conditions, then after 10, 20, and 40 minutes of heat shock (HS). For the DMSO and resveratrol groups, nuclei were imaged at 10, 20, and 40 minutes post-treatment under NHS, followed by a 10-minute HS after 50 minutes of total treatment time. For the untreated positive control group, 4 biological replicates (individual salivary glands) were analyzed with 4 nuclei imaged per replicate (total 16 nuclei). For the 0.5% DMSO group, 4 biological replicates were analyzed with 4 nuclei imaged per replicate (total 16 nuclei). For the 100 µM resveratrol group, 4 biological replicates were analyzed with 4 nuclei imaged per replicate (total 16 nuclei). For the 200 µM resveratrol group, 5 biological replicates were analyzed with 3, 4, 2, 2, and 5 nuclei imaged from replicates 1-5, respectively (total 16 nuclei). For the 400 µM resveratrol group, 4 biological replicates were analyzed with 4, 4, 3, and 3 nuclei imaged from replicates 1-4, respectively (total 14 nuclei). Red arrows indicate HSF-GFP localization to the Hsp70 loci. Scale bar: 10 µm. C.) Western blot analysis of Hsp70 protein expression in salivary glands from male transgenic GFP-HSF *Drosophila melanogaster* under different treatment conditions. The top panel shows a representative blot probed for Hsp70 and tubulin. The bottom panel presents densitometric analysis of Hsp70 levels normalized to tubulin, averaged from three biological replicates. Samples 1-2 represent the negative control group: flies treated for 20 minutes (1) without or (2) with 0.5% DMSO under non-heat shock (NHS) conditions, followed by 45 minutes of recovery in NHS conditions without DMSO. Samples 3-4 are the positive control group: flies exposed to 20 minutes of heat shock (3) without or (4) with 0.5% DMSO, then allowed to recover for 45 minutes under NHS conditions without DMSO. Samples 5-7 were treated with 100 µM, 200 µM, or 400 µM resveratrol (dissolved in 0.5% DMSO) for 20 minutes under NHS conditions, followed by 45 minutes of recovery in NHS conditions without resveratrol or DMSO. All recovery steps were included to allow time for Hsp70 protein accumulation. Each experimental group was analyzed using three independent biological replicates, with lysates prepared from 15 salivary glands per replicate. Error bars represent 95% confidence intervals. One-way ANOVA followed by Tukey’s post hoc test showed that Hsp70 levels were significantly elevated in heat-shocked flies compared to all other groups (**p < 0.01 to ****p < 0.0001). No significant differences were observed between the two heat-shock groups (with or without DMSO), or between resveratrol-treated and non-heat-shocked controls.

## Description

Proteins drive most of the biological process in cells. Thus, environmental stresses, like heat shock (HS), which cause loss of protein structure and function, threaten the viability of organisms. To overcome this issue, cells utilize a highly conserved stress response pathway, called the heat shock response (HSR) (Akerfelt et al., 2010). HSR is initiated when the transcription factor heat shock factor (HSF) binds to the *hsp70* loci, triggering transcription of the gene and ultimately the production of the Hsp70 protein (Akerfelt et al., 2010). The Hsp70 protein exhibits a multitude of fundamental intracellular roles that are necessary for cell survival, such as chaperone activities that result in stabilization and protection of newly synthesized proteins (Rosenzweig et al., 2019).

Heat stress also increases the amount of reactive oxygen species (ROS), which can damage proteins through oxidative stress (Belhadj Slimen et al., 2014). ROS are naturally synthesized within the body through several cellular systems, including within the plasma membrane, the cytosol, and in the membranes of the mitochondria and endoplasmic reticulum (Di Meo et al., 2016). While locally made in the body, if ROS are in excess, oxidative damage can occur. Denaturation of proteins via excessive ROS production usually occurs through protein carbonylation, tyrosine nitration, and reversible cysteine modification (Kehm et al., 2021).

Under normal cellular conditions, the amount of ROS is readily controlled by antioxidants as they scavenge and destroy/neutralize free radicals through redox reactions (Sharifi-Rad et al., 2020). When these reactions occur, antioxidants are oxidized, rendering the antioxidant incapable of reacting with more free radicals and, thus, requiring antioxidant concentrations to continually be replenished. Notably, antioxidants can be obtained both endogenously and exogenously. One exogenous antioxidant is resveratrol, which is commonly found in red wines, along with other phenolics, and contains strong antioxidant properties (Pezzuto, 2019). Supplementation with resveratrol has been reported in some human clinical studies to mitigate health complications related to oxidative stress, including neurodegenerative and cardiovascular diseases. However, findings from clinical studies are mixed, with some showing beneficial effects and others reporting no significant impact (Pezzuto, 2019).

Notably, there are also conflicting studies of the effects of resveratrol on the health of *Drosophila melanogaster* . While some research indicates that resveratrol does not enhance the flies' resistance to oxidative stress (Staats et al., 2018), other studies suggest that it may confer protective effects against oxidative stress under specific conditions (Abdulazeez et al., 2024; Abolaji et al., 2019; Bonilla et al., 2012; Danilov et al., 2015; Wang et al., 2013). These discrepancies underscore the need for further investigation into the role of resveratrol in *Drosophila* stress biology.

Prolonged high heat shock at 39°C causes adult *Drosophila* to undergo paralysis (Gong & Golic, 2006). Thus, to better understand the role of resveratrol in stress responses, we tested its impact on the kinetics of paralysis induced by high heat shock in wild-type Canton-S adult *Drosophila* . Flies were fed resveratrol-supplemented food for five days prior to the heat stress assay. Notably, pretreated male flies given 100 μM, 200 μM, and 400 μM resveratrol showed a significant increase in the time it took for high heat shock to induce paralysis compared to untreated flies (Figure 1a). These concentrations are based on prior studies discussed above, where the resveratrol was commonly administered to *Drosophila* in the 10–500 μM range (Abdulazeez et al., 2024; Abolaji et al., 2019; Bonilla et al., 2012; Danilov et al., 2015; Staats et al., 2018; Wang et al., 2013). However, resveratrol did not show a significant effect in females (Figure 1a). This is not surprising, as sex-specific effects have been observed in *Drosophila* with various antioxidants, including resveratrol. (Danilov et al., 2015; Wang et al., 2013). Our results indicate that in males, resveratrol may possess properties that help prevent reactive oxygen species (ROS) from damaging proteins during heat stress. Previous studies have shown that certain chemicals can modulate the heat shock response (HSR), including promoting heat shock factor (HSF) binding to chromatin under specific conditions in both human and *Drosophila* cells (Jurivich et al., 1992; Winegarden et al., 1996). Thus, we hypothesized that resveratrol’s protective effects may not be solely due to its antioxidant activity, but also due to its potential to activate the HSR in male flies, thereby priming them to better resist heat shock-induced paralysis.

To begin to test this hypothesis, we used a well-established live-cell imaging system based on *Drosophila* salivary glands, which contain cells with polytene chromosomes that enable confocal visualization of HSR activation (Yao et al., 2006; Choi et al., 2018). This system utilizes salivary glands from transgenic *Drosophila* third instar larvae expressing green fluorescent protein-tagged HSF (GFP-HSF). The polytene chromosomes within these cells are gigantic interphase chromosomes that allow for clear visualization of transcription factor-gene loci interactions. Heat shock of the glands at 37°C for 10, 20, and 40 minutes triggers HSF-GFP to bind robustly to the Hsp70 loci on the polytene chromosomes. This recruitment can be observed as a characteristic doublet of green bands at cytological sites 87A and 87C (indicated by red arrows in the top panel of Figure 1b). The 87A band contains two copies of the Hsp70 gene, and 87C contains four. Importantly, this system also allows us to test whether resveratrol alone, in the absence of heat, is sufficient to trigger HSF recruitment and activate the stress response in male flies.

For both the initial heat shock controls described above and the resveratrol experiments described below, salivary glands were isolated from male larvae. For the resveratrol experiments, glands were incubated for 10, 20, and 40 minutes with either 100 μM, 200 μM, or 400 μM resveratrol dissolved in DMSO, or with DMSO carrier alone, and then imaged to assess HSF-GFP localization at the Hsp70 loci (bottom panel of Figure 1b). Glands treated with resveratrol or DMSO alone showed no detectable HSF-GFP recruitment to the Hsp70 loci at any time point. For each condition, a 10-minute heat shock was applied after the 40-minute incubation with resveratrol or DMSO, resulting in robust HSF-GFP localization to the Hsp70 loci. Together, these live-cell imaging results demonstrate that under these conditions, neither DMSO nor resveratrol activates or impairs the HSR pathway.

To complement the live-cell imaging experiments, we used a molecular approach to evaluate whether resveratrol activates the HSR pathway at the protein level. As shown in Figure 1c, salivary glands from male larvae were treated for 20 minutes under six conditions: non-heat shock (with or without DMSO), heat shock (with or without DMSO), and resveratrol at 100 μM, 200 μM, or 400 μM dissolved in DMSO. Western blotting was performed on protein lysates from each treatment to assess Hsp70 protein levels, with tubulin serving as a loading control. The top panel of Figure 1c displays a representative western blot, while the bottom panel shows densitometry quantification based on the average of three independent biological replicates. Samples without heat shock, both with and without DMSO, showed low Hsp70 levels. In contrast, heat-shocked samples displayed significantly elevated Hsp70 expression, regardless of DMSO presence, confirming robust activation of the HSR pathway. Importantly, glands treated with 100 μM, 200 μM, or 400 μM resveratrol exhibited Hsp70 levels statistically indistinguishable from the non-heat-shocked DMSO control group, indicating that resveratrol alone does not induce Hsp70 protein expression. These data support the conclusion that resveratrol does not activate the HSR under these conditions. Taken together, the imaging and molecular results consistently demonstrate that resveratrol does not activate the HSR pathway under these experimental conditions.

These findings suggest that resveratrol’s protective effects in male flies are likely mediated through an HSR-independent mechanism. In addition, resveratrol is known to exert pleiotropic cellular effects, including DNA damage responses and anti-inflammatory activities that may involve post-transcriptional regulation of mRNA stability (for example, destabilization of certain stress-related transcripts) (Bollmann et al., 2014). While such mechanisms could, in principle, influence Hsp70 expression, our results show no evidence of HSF recruitment or Hsp70 induction, suggesting that resveratrol does not activate the heat shock response under the conditions tested. Instead, the observed benefits may arise from resveratrol’s antioxidant properties. However, multiple stress response pathways beyond the HSR regulate cellular protein quality control, including the unfolded protein response (UPR) (Fulda et al., 2010). The unfolded protein response (UPR) is a cellular stress pathway that mitigates protein misfolding in the endoplasmic reticulum caused by various stresses, including oxidative stress. Notably, resveratrol has been shown to alleviate endoplasmic reticulum (ER) stress (Ahmadi et al., 2021). These findings raise the intriguing possibility that resveratrol mitigates heat stress paralysis via alternative mechanisms, including, but not limited to, the UPR. Investigating whether resveratrol activates these other pathways represents a promising direction for future research

## Methods

*Drosophila melanogaster* strains:

Wild-type Canton-S flies were obtained from Indiana University Bloomington *Drosophila* Stock Center (Stock number 64349). GFP-HSF transgenic flies were a generous gift from the Lis lab (Cornell University), originally described in Yao et al. (2006).

Heat shock paralysis assay:

*Drosophila* food (Genessee Scientific, 66-112) in plastic vials with cotton stoppers was supplemented with either 0.5% DMSO (Sigma-Aldrich, 34869) or 100 μM, 200 μM, or 400 μM resveratrol (Sigma-Aldrich, R5010) dissolved in 0.5% DMSO. Wild-type Canton-S flies were incubated at room temperature on food with or without resveratrol for 5 days. Flies were transferred to fresh food with the respective chemical treatments daily. On day 5, heat shock paralysis was assessed by transferring flies to empty plastic vials and submerging the vials into a water bath set to 39°C to heat the air inside. A timer was used to record the time until each fly became paralyzed. Statistical analysis was performed in GraphPad Prism (Prism Software).

Live-cell imaging assay:

Salivary glands from male third instar GFP-HSF transgenic *Drosophila melanogaster* larvae were dissected in a PYREX nine-well spot plate (Corning, 722085) containing room temperature 5/6 Grace’s Insect Medium (Thermo Fisher Scientific, 11605094). Male larvae were identified by the presence of prominent gonad discs. For the initial HS control, glands were transferred to a MatTek glass-bottom dish (MatTek, P35G-1.5-14-C) containing room temperature Grace’s medium. Samples were first imaged with a room temperature 40x oil immersion objective (Olympus, UPLXAPO 40X Objective) using a laser scanning confocal microscope (Olympus, FLUOVIEW 3000). To induce heat shock, a matching 40x oil immersion objective pre-heated to 37 °C (using the Bioptechs objective heater (Bioptechs, 50803/150819-19) was applied directly to the sample for 10, 20, and 40 minutes, with confocal images captured after each time point. For the resveratrol experiments, dissected salivary glands were transferred to MatTek glass-bottom dishes containing either 0.5% DMSO or 100 μM, 200 μM, or 400 μM resveratrol dissolved in 0.5% DMSO. Samples were imaged using a room temperature 40x oil immersion objective at 10, 20, and 40 minutes post-treatment. After the final time point, heat shock was induced by applying a matching 40x oil immersion objective pre-heated to 37 °C, and confocal images were captured after 10 minutes of heat shock. Maximum intensity confocal microscopy images and scale bar were generated using the imaging software ImageJ (National Institutes of Health).

Western blot assay:

Salivary glands were dissected from male third instar GFP-HSF transgenic *Drosophila melanogaster* larvae in room temperature 5/6 Grace’s Insect Medium using a PYREX nine-well spot plate. Male larvae were identified by the presence of prominent gonad discs. In the PYREX dish, glands were subjected to the following treatment conditions (numbers in parenthesis correspond to sample numbers in Figure 1C ): (1) non-heat shock (NHS) in medium alone, (2) NHS in medium containing 0.5% DMSO, (3-4) heat shock (HS; 20 min at 37 °C) in medium with or without 0.5% DMSO, and (5-7) NHS in medium containing 100 μM, 200 μM, or 400 μM resveratrol dissolved in 0.5% DMSO. Heat shock was performed using pre-warmed medium in a 37°C incubator. All treatments were followed by a 45-minute recovery period under NHS conditions in Grace’s medium lacking DMSO or resveratrol to allow Hsp70 protein accumulation. For each condition, approximately 15-20 salivary glands were pooled. Total soluble protein was extracted using RIPA buffer (Thermo Fisher Scientific, 89900) supplemented with Halt Protease Inhibitor Cocktail (Thermo Fisher Scientific, PI78437). The entire lysate from 15 salivary glands per well was loaded and analyzed by western blot. Chameleon Duo Pre-stained Protein Ladder (LI-COR Biosciences, 928-60000) was used as a molecular weight marker. Western blotting was performed using the Bio-Rad Mini-PROTEAN TGX system with 12% precast gels (Bio-Rad, 4561044) and Trans-Blot Turbo nitrocellulose membranes (Bio-Rad, 1704158), following the manufacturer’s instructions. Membranes were incubated with the following primary antibodies: mouse anti-Hsp70 (1:1,000; Thermo Fisher Scientific, MA3-007) and rabbit anti-α-tubulin (1:5,000; Cell Signaling Technology, 4967L). Membranes were incubated with the following secondary antibodies: IRDye 680RD goat anti-mouse IgG (1:10,000; LI-COR Biosciences, 926-68070) and IRDye 800CW goat anti-rabbit IgG (1:10,000; LI-COR Biosciences, 926-32211).Membranes were imaged using the LI-COR Odyssey QF imaging system (LI-COR, Odyssey QF imaging system), and band intensities were quantified using ImageJ software (National Institutes of Health). Tubulin was used as a loading control to normalize relative levels of Hsp70 protein. Statistical analysis was performed in GraphPad Prism (Prism Software).
